# Winning the war against ICU-acquired weakness: new innovations in nutrition and exercise physiology

**DOI:** 10.1186/cc14724

**Published:** 2015-12-18

**Authors:** Paul E Wischmeyer, Inigo San-Millan

**Affiliations:** 1 Department of Anesthesiology, University of Colorado School of Medicine, 12700 E. 19th Avenue, Box 8602, RC2 P15-7120, Aurora, CO 80045, USA; 2 Department of Physical Medicine & Rehabilitation, University of Colorado, School of Medicine, Aurora, CO, USA

## Abstract

Over the last 10 years we have significantly reduced hospital mortality from sepsis and critical illness. However, the evidence reveals that over the same period we have tripled the number of patients being sent to rehabilitation settings. Further, given that as many as half of the deaths in the first year following ICU admission occur post ICU discharge, it is unclear how many of these patients ever returned home. For those who do survive, the latest data indicate that 50-70% of ICU "survivors" will suffer cognitive impairment and 60-80% of "survivors" will suffer functional impairment or ICU-acquired weakness (ICU-AW). These observations demand that we as intensive care providers ask the following questions: "Are we creating survivors ... or are we creating victims?" and "Do we accomplish 'Pyrrhic Victories' in the ICU?" Interventions to address ICU-AW must have a renewed focus on optimal nutrition, anabolic/anticatabolic strategies, and in the future employ the personalized muscle and exercise evaluation techniques utilized by elite athletes to optimize performance. Specifically, strategies must include optimal protein delivery (1.2-2.0 g/kg/day), as an athlete would routinely employ. However, as is clear in elite sports performance, optimal nutrition is fundamental but alone is often not enough. We know burn patients can remain catabolic for 2 years post burn; thus, anticatabolic agents (i.e., beta-blockers) and anabolic agents (i.e., oxandrolone) will probably also be essential. In the near future, evaluation techniques such as assessing lean body mass at the bedside using ultrasound to determine nutritional status and ultrasound-measured muscle glycogen as a marker of muscle injury and recovery could be utilized to help find the transition from the acute phase of critical illness to the recovery phase. Finally, exercise physiology testing that evaluates muscle substrate utilization during exercise can be used to diagnose muscle mitochondrial dysfunction and to guide a personalized ideal heart rate, assisting in recovery of muscle mitochondrial function and functional endurance post ICU. In the end, future ICU-AW research must focus on using a combination of modern performance-enhancing nutrition, anticatabolic/anabolic interventions, and muscle/exercise testing so we can begin to create more "survivors" and fewer victims post ICU care.

## Introduction

Another such victory ... and we shall be undone.

(Pyrrhus of Epirus)

Over the last 10 years we have significantly reduced mortality following sepsis and critical illness [1]. Upon hearing these data most ICU clinicians feel the urge to "high-five" their colleagues and believe we are winning the battle against critical illness. But this raises the key question: "Are we winning many battles in the ICU, but ultimately losing the war?" Are the battles we are winning really just "Pyrrhic Victories"? The history of this statement, and perhaps an eye-opening lesson for us as critical care practitioners, harkens back to the early days of the Roman Empire (281 BC) when the small Greek city of Tarentum was threatened with attack by Roman forces. The small city appealed to the great Greek general Pyrrhus to save them from the Romans. Pyrrhus, a descendent of Alexander the Great, came to their aid, landing in Italy with an army of 3000 cavalry, 2000 archers, 500 slingers, 20,000 infantry, and 20 war elephants in the attempt to subdue the Romans. He proceeded to defeat the Romans at Battle of Heraclea in 280 BC. In victory, however, Pyrrhus lost 13,000 of his best soldiers, nearly half of his original force. The following year, Pyrrhus and the Romans faced off again in the Battle of Asculum. Pyrrhus won what proved to be a backbreaking victory but losing many thousands of additional men, including most of his officers. Following the battle, Pyrrhus famously stated (paraphrase--many translations exists) "Another such victory and we shall be utterly ruined". It is from reports of this semi-legendary event that the term "Pyrrhic victory" originates. Pyrrhus continued to fight in Italy and ultimately in Sicily where he took on the empire of Carthage while also fighting the Romans. He continued to prove victorious in every battle, but always with heavy losses. Ultimately, despite never losing a battle, Pyrrhus was forced to retreat to Greece with only 8000 infantry and 500 cavalry, with nothing to show for it but a depleted treasury. Although he initially escaped with his life, just 2 years later in a battle with Sparta Pyrrhus was hit on the head by a roof tile thrown by an elderly townswoman. Stunned by the blow, he became disoriented and was beheaded by a Macedonia soldier. Although listed by Hannibal as perhaps the greatest general to ever live, Pyrrhus is now only remembered as a general who won many battles but ultimately lost his most important war.

What can Pyrrhus teach us as ICU providers? We must consider that in critical care we are also winning many battles in our ICUs. As we have reduced mortality from sepsis by half [1], we have apparently learned to win a number of battles, including the resuscitation battle with fluids and vasopressors [2], and we have begun to win the ventilator-associated injury/acute respiratory distress syndrome (ARDS) "battle" because mortality from ARDS in the control group of a recent large ARDSnet trial was down to a startling 16% [3]. Despite these successful practices, the same data which indicate we have reduced sepsis hospital mortality by half in the last 10 years also show that we "tripled the number of patients going to rehabilitation" [1]. This begs the following question: of these "ICU survivors", how many even survived a year? Troubling data over the last 10-15 years show that as much as 40-50% of the mortality within 12 months of an ICU admission occurs after the patient leaves the unit [4]. Commonly, patients are placed in a nursing home or a rehabilitation center, never to return home to their loved ones or to hold their grandchildren again. Thus, authors from many leading critical care trials groups are stating: "Given low ICU mortality ... quality of life, not mortality should become the focus of future large ICU trials" [1]. More practically, for all of us as ICU caregivers, we all must ask ourselves: "Are we creating survivors ... or victims" in our ICU care?

## "Are we creating survivors ... or victims?"

To answer this question, perhaps we need to be better at asking our patients what they think about their quality of life (QOL) following their stay in our ICUs. One of the leaders in this field, Dr Wes Ely and his group at Vanderbilt University have begun to ask these questions of patients, and his group has created a website [5] for ICU patients and their families that we as caregivers can gain great insight from. One of these interviews is with a middle-aged woman named Melissa who had previously survived a 2-year battle with leukemia. Unfortunately, many years later Melissa was diagnosed with influenza pneumonia, which unfortunately evolved to ARDS and led to an ICU stay requiring mechanical ventilation. Following her ICU stay, Dr Ely and his group interviewed Melissa and her husband about their experience with ARDS and recovery from critical illness. In her own words, Melissa compares her 2-year experience with leukemia and chemotherapy with her brief experience with ARDS in the ICU. In a poignant moment she states: "I never dreamed after having had leukemia and done two years with chemo ... I never dreamed that anything else could be worse ... and this was so much worse. It was more spiritually, emotionally, physically, intellectually challenging than even cancer ... *if you presented me ARDS and leukemia ... I would choose the leukemia*" (see Additional file 1). This is a statement that should send chills down the spine of those of us who have committed our lives to the care of ICU patients. Undoubtedly we are winning the battle when it comes to being able to save patients at any cost and get them "out of the ICU", but are we winning the war, especially when it comes to post-ICU QOL? Melissa goes on to describe her QOL following her ICU stay as she states "I was so weak I could hardly lift my limbs off the bed, I could not sit up, and when they got me into an upright position it was absolutely terrifying ... I couldn't walk, I couldn't stand ... I had to learn how swallow." Her husband adds: "I remember when ... the doctors told me it will be several *months *in rehab, and I was like ... but this is only pneumonia? ..." (see Additional file 2). Of course, we know from the work of Margaret Herridge [6-8] and others that Melissa's experience is not unique. Dr Herridge and her colleagues have shown that even 40-year-old and 50-year-old ICU patients report median Short Form-36 (SF-36) physical QOL scores of 0 at 3 and 6 months following an ICU stay for ARDS [6-8]. This marked impairment in QOL persists for 1 year and often even more than 5 years, as shown in her subsequent work. We know that 50% of these patients are not back at work at 1 year and one-third will never return to work [6-8]. Overall, recent data indicate that cognitive impairment will affect 50-70% [9] of our ICU patients and 60-80% [10] will be functionally impaired post ICU. Without doubt, this is an epidemic. The question must then become what can we do to start winning this war?

## Why are we losing the quality of life war post ICU?

Recent research indicates that the critically ill burn patient can lose as much as 1 kg lean body mass per day [11]. Other critically ill patients also suffer significant lean body mass loss, much of it in the first 7-10 days of their ICU stay [11]. Patients will gain weight back following their ICU stay, but virtually all of this weight is fat mass, not functional lean body mass [12]. This is not surprising, as data from burn ICU patients demonstrate that the catabolic/hypermetabolic state following injury can persist for up to 2 years following discharge from the hospital and this can markedly hinder recovery of patients' lean body mass and function following injury [13,14]. Further, we as ICU caregivers have not learned to extend our care beyond the ICU's borders, such as via post-ICU rounds and ICU recovery clinics. Given that patients must be taught how to manage post-ICU regimens such as insulin control of hyperglycemia, why should we expect patients to be able to intuitively know how to regain lean body mass and function, especially in the face of a persistent hypermetabolic/catabolic state? Many fully healthy individuals and elite athletes pay millions of dollars each year to professional athletic trainers to "teach" them how to lose fat mass, gain lean body mass, and improve their physical performance. Who needs this more than our ICU patients?

## Are checklists and bundles the answer after the ICU?

**Figure 1:**
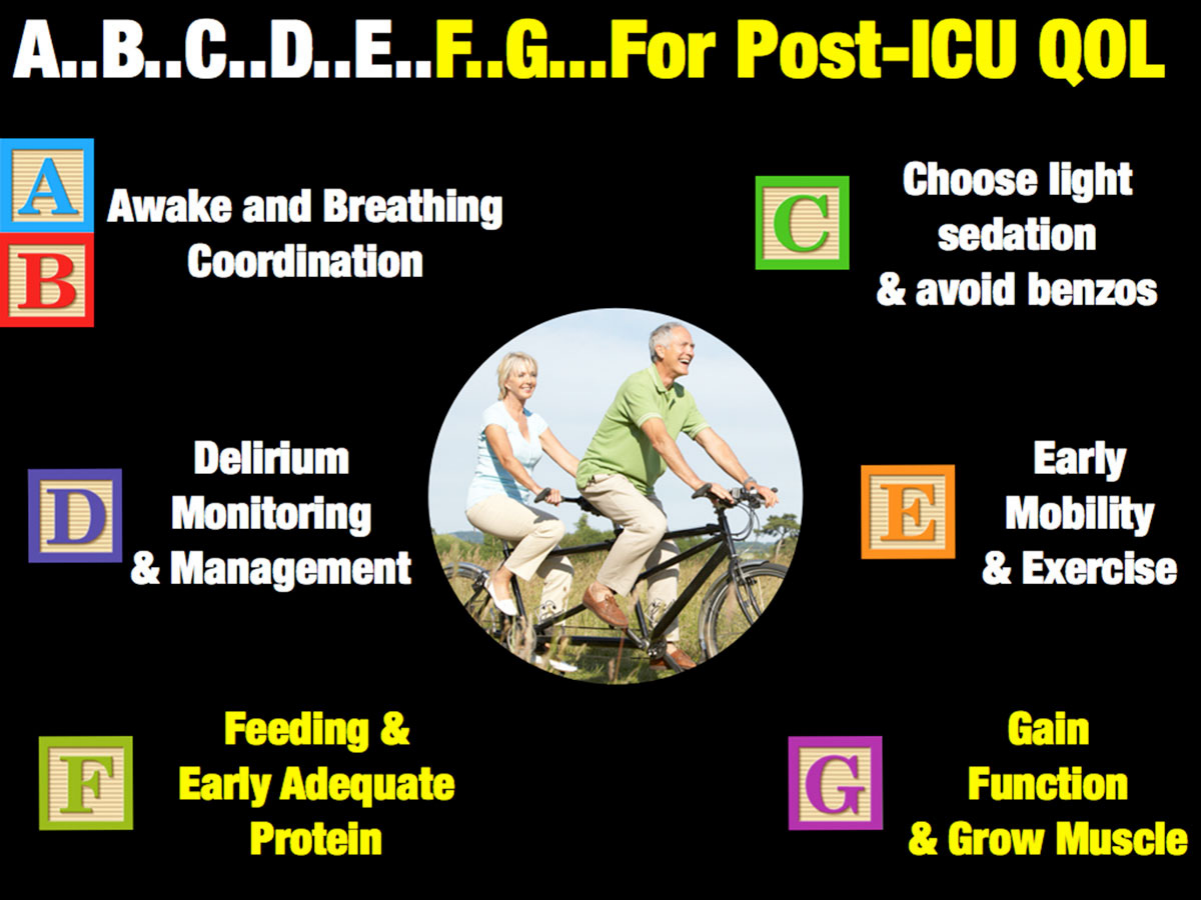
**A ... B ... C ... D ... E ... F ... G ... for post-ICU QOL.*QOL *quality of life**.

In ICU care we have checklists and bundles for virtually all of our care. We have guidelines, such as the Surviving Sepsis Campaign, and pneumonics like "FAST-HUG" (Feeding, Analgesia, Sedation, Thromboembolic Prophylaxis, Head-of-Bed Elevation, Ulcer Prevention, Glucose Control) for in-ICU care [15]. In improving post-ICU QOL we have recently been introduced to the ABCDE bundle [16] for improving long-term outcomes (see Figure 1). We would advocate that we should add F and G, with F emphasizing the basic need for Feeding and early adequate protein and G emphasizing the role for Gaining function and Growing muscle. ABCDE has been well described by Dr Ely and others [16], but how do we achieve the F and the G and perform the research necessary to optimize these key parts of future ICU care?

Our research group is developing an ICU recovery program known as "RISEN"--Recovery from ICU via Surveillance, Exercise, and Nutrition. This protocol attempts to learn and study techniques and practices utilized by elite athletes, such as Tour de France cyclists and competitive strength athletes, to benefit our patients' battle to become ICU survivors and not ICU victims. This experimental protocol (Table 1) proposes that in order to optimize post-ICU QOL we must first evaluate the nutritional needs of our patients via accurate metabolic cart caloric need measurements (so as to prevent underfeeding and overfeeding). The era of "guessing" at caloric needs with equations must end--we would not guess at the blood pressure while administering vasopressors, so why should we guess at caloric needs when the epidemic of obesity and the increasingly older ICU patient have proven convincingly that we are not good "guessers" [17]. Further, we need to estimate nutritional risk with scores such as the NUTRIC score [18], so we know in whom nutrition is more urgently needed; for example, by supplemental parenteral nutrition (PN) to complement enteral feedings. Three very recent large trials have shown PN to be safer than ever and not associated with high incidence of infection, as in years past [19-21].

**Table 1:** Recovery from ICU via Surveillance, Exercise, and Nutrition (RISEN) protocol

**Day 1 (ICU admission)**
Baseline nutritional and metabolic evaluation
Nutrition risk score (NUTRIC score?)
Metabolic evaluation with indirect calorimetry
Baseline muscle evaluation
Lean body mass ultrasound
Muscle glycogen ultrasound
Markers of muscle injury?
CPK-MM (creatine phosphokinase-muscle component)
Myoglobin
LDH-(lactate dehydrogenase)
**Every 3-7 days post ICU admission**
Ongoing nutritional evaluation
Indirect calorimetry to guide feeding and assess recovery and mitochondrial function along with lactate measurements.
Muscle evaluation
Lean body mass ultrasound
Muscle glycogen ultrasound
Markers of muscle injury?
CPK-MM
Myoglobin
Physiologic exercise evaluation (when able)
Diagnose exercise/mitochondrial function
Individualized exercise prescription including resistance training to improve metabolic and musculoskeletal function.

We next need to implement and evaluate bedside lean body mass and muscle function, perhaps using tests such as the lean body mass ultrasound and muscle glycogen ultrasound. Recent data have begun to show that the use of routine ultrasound techniques (the same ultrasound used to place central lines present in virtually all ICUs worldwide) can accurately predict lean body mass [22]. These relatively simple tools appear to be as accurate as magnetic resonance imaging (MRI) or computed tomography (CT) scan lean body mass estimation in patients and healthy subjects [22]. This technology is currently being validated in ICU patients, and we have shown it has excellent inter-rater and intra-rater reliability when performed by almost any caregiver including dieticians and nursing staff [23]. This may finally provide an objective method to identify sarcopenic patients at ICU admission--those, we believe, who are most at risk for mortality and impaired post-ICU QOL from poor pre-ICU lean body mass reserve. Such information will also guide us in determining who should receive early, aggressive protein and calorie delivery (via PN and/or enteral nutrition (EN)) in both clinical trials and clinical practice. We believe this method will also allow objective evaluation of the effect of our nutrition and early mobility efforts at the bedside to reduce loss of lean body mass and, hopefully, its recovery [22]. Understanding these processes is vital, as currently we know that patients who lose 40% of their lean body mass have an exceedingly high mortality, with much of this mortality related to lost lean body mass occurring post ICU discharge [11].

**Figure 2:**
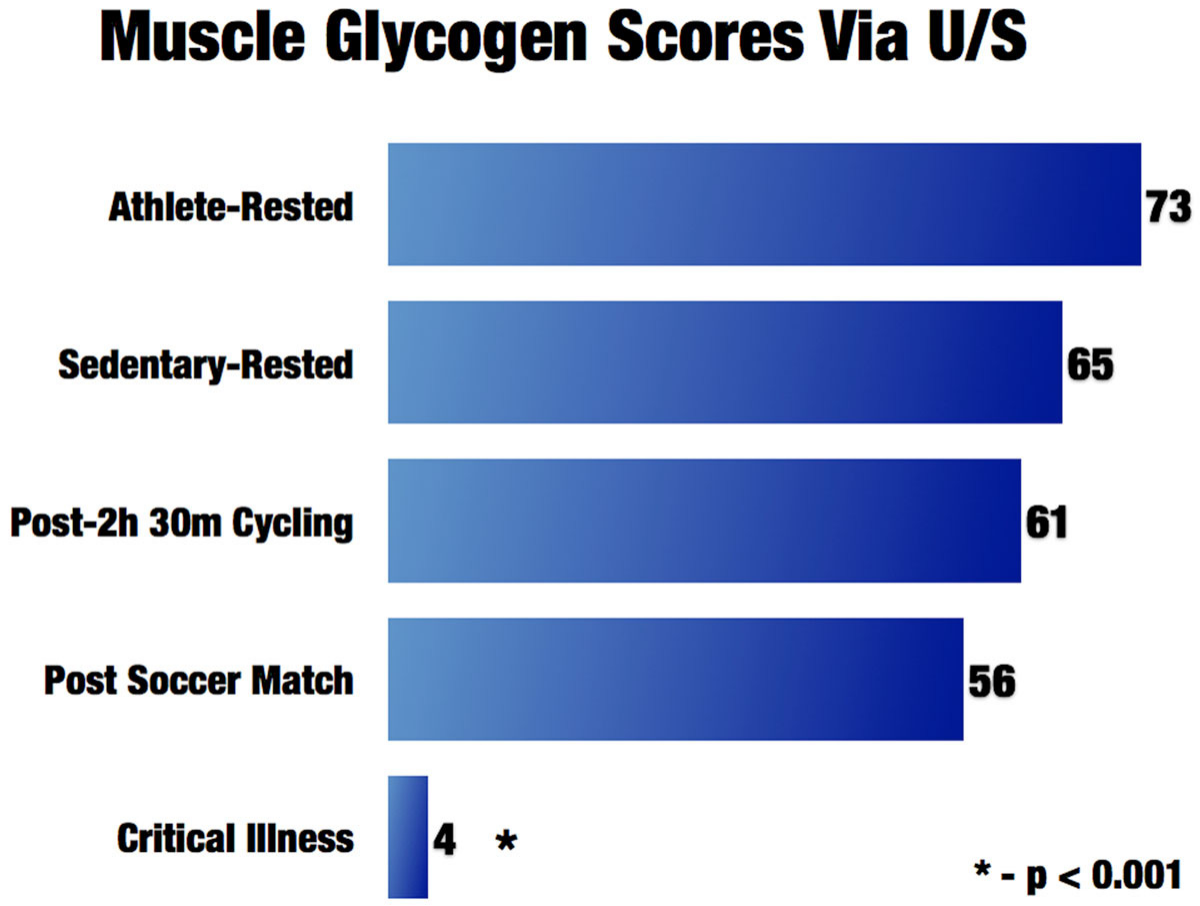
**Muscle glycogen scores via ultrasound**.

**Figure 3:**
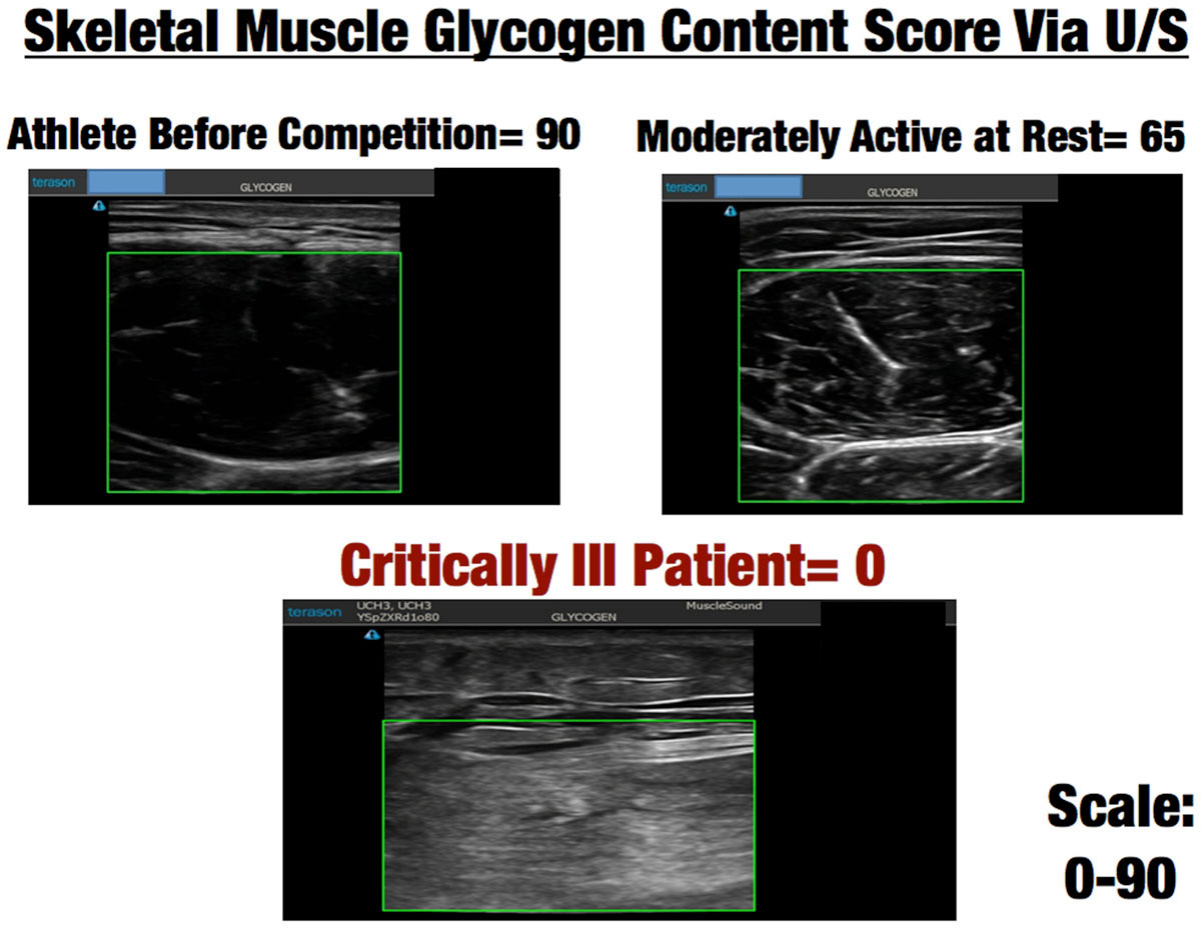
**Skeletal muscle glycogen content score via ultrasound**.

Further, new cutting-edge ultrasound technology is being utilized by the world's most elite cyclists in the Tour de France and other professional sports to directly measure muscle glycogen within a few minutes [24]. This proven muscle biopsy technique evaluates the glycogen storage of the muscle [24]. This technology is utilized by athletes to predict and prevent overtraining and guide nutritional needs during training. Glycogen depletion leads to marked muscle damage and an inability for muscle to recover and become anabolic, as muscle protein must be broken down for energy when energy cannot be obtained from glycogen stores. This leads to ongoing catabolism and inability to recover muscle mass and function [24]. This technique scores muscle glycogen content on a scale of 0-90, with 90 being optimal or peak muscle glycogen content in healthy muscle. We have evaluated numerous athletes and found that the average athlete who is rested and well nourished will have an average muscle glycogen score of 73. Following 2 hours and 30 minutes of strenuous cycling or running a marathon, the score drops to 50-60 (see Figure 2). We used this muscle glycogen ultrasound technology to assess nine ICU patients at varying points in their ICU stay as part of our ICU nutrition program evaluation; that is, in order to carry out quality assessment of our ICU nutrition delivery at University of Colorado (not research as defined by the US Department of Health and Human Services). The muscle glycogen score averaged 4, with seven of the nine patients scoring 0, which we have never observed in any other human population [25]. Thus, one might equate being in the ICU as similar to continuously running multiple marathons. We are currently validating this finding in ICU patients with muscle biopsies to confirm the previous muscle biopsy validation data performed in healthy volunteers. Examples of ultrasound pictures obtained from athletes, sedentary individuals, and an ICU patient are shown in Figure 3.

## What role does nutrition and protein delivery play in post-ICU outcomes?

Without doubt, lean body mass preservation and recovery of muscle mass and function following critical illness cannot be achieved without optimal protein and calorie delivery. Great controversy has arisen recently between two differing opinions regarding optimal feeding in the ICU. Traditionally, it has been advocated that patients receive 80% of full calorie and protein (1.2-2.0 g/kg/day) feeds in the first week of ICU to optimize outcomes [26]. However, a number of recent trials have advocated "trophic feeding" or intentional underfeeding in the first ICU week, suggesting equal efficacy [27-29]. However, it is intuitive to most ICU practitioners that "all ICU patients are not created equal" and undoubtedly "one size does not fit all". This concept is well described in a recent publication from Daren Heyland's research group demonstrating that high-risk ICU patients (mechanically ventilated >8 days) who received low nutritional adequacy in the first week of their ICU stay (<50% of predicted caloric need) presented increased mortality (adjusted hazard ratio (HR) = 1.7, 95% confidence interval (CI): 1.1-2.6) versus patients receiving high nutritional adequacy (>80% of calorie needs) after key covariate adjustment [30]. These data also demonstrate that for every 25% increase in calorie delivery in the first ICU week, an improvement in 3-month post-ICU physical QOL scores (as measured by the SF-36) is observed. In medical ICU (MICU) patients (typically found to have greater preillness comorbidities) the effect of improved nutritional adequacy on QOL was much stronger with significant improvements in both 3-month and 6-month SF-36 scores [30]. Importantly, these QOL improvements are greater than the minimum clinical important differences (CIDs) for pulmonary disease [31], found to be meaningful in a patient's perceived QOL. Experts in the ICU QOL field have extrapolated these CIDs in pulmonary disease to post-ICU QOL because no CIDs for critical illness have been established [32]. CIDs for pulmonary disease are described as a change of ≥10 on the SF-36 scale for physical functioning and a ≥12.5 point change for role-physical [31]. The data presented from this recent publication show that for every 25% increase in caloric delivery over the first 8 days in the MICU, there is a 10.9 point increase in physical functioning and a 13.1 point increase in role-physical measures. Thus, a 50 or 75% increase in caloric delivery over the first week of stay in the MICU would be expected to lead to a 20-30 point change in physical functioning and a 26-40 point change in role-physical. These changes would equate to a large change in perceived QOL for ICU patients following discharge [31]. A recent trial by the ANZIC's group has indicated that a 7.8 point change in physical QOL domain scores is considered clinically relevant based on pilot trial data. These data thus indicate clinically significant changes in post-ICU QOL may be achieved even with a 25% increase in caloric delivery during the first 8 days of the ICU stay [33].

A major differentiating factor in randomized clinical trials showing benefit on clinical outcome from reaching goal nutrition delivery (Table 2) versus trials not demonstrating a benefit of reaching goal nutrition is that all trials showing benefit on clinical outcomes reached a protein delivery of >1.0 g/kg/day in the higher nutrition delivery group. [19,20,27-30,52,53]

**Table 2:** Comparison of Outcomes and Differences of Recent Nutrition Delivery Trials in ICU2.

	Trials not supporting goal (>80% kcal/day) nutritional delivery in the ICU				Trials supporting goal (> 80% kcal/day) nutritional delivery in the ICU			
	
	EPaNIC (*NEJM*, 2011) (BB)	EDEN Trial (Pilot) (*CCM*, 2011) (29)	Eden Trial (Full RCT) (*JAMA*, 2012) (28)	Arabi Trial (*AJCN*, 2011) (27)	Early PN (*JAMA*, 2013) (19)	TICACOS (*ICM*, 2011) (AA)	SPN (*Lancet*, 2013) (20)	Wei et al. (*CCM*, 2015) (30)
Age (mean)	64	53	52	51	69	61	61	62
ICU LOS	3.5			13.1	9	12	13	18
Hospital LOS	15				25	25	31.5	
MV days	2	5.6	5	11.9	6.9	10.75	6.64	15
Mortality (%)								
ICU	6.2			19.6		25.4	10	26
Hospital	10.65	21		36	21%	38.3	24	32
Post discharge	11.2		22.7	38.6		47	23	
Primary outcome	Sig. reduced LOS in ICU for late PN (median 3 days) vs. early PN (median 4 days)	No outcome changes in trophic versus full feeding groups for ventilation days, mortality, or infection	No outcome changes in trophic versus full feeding groups for ventilation days, mortality, or infection	Nonsig. trend to lower 28-day mortality for trophic (18.3%) compared with target feeding (23.3%) (*p *<0.07)	No sig. change in crude day-60 mortality (standard care (22.8%) vs. early PN (21.5%))	Sig. lower hospital mortality for goal calorie group (28.5%) vs. underfed control group (48.2%)	Sig. reduced nosocomial infections for EN + SPN (27%) vs. EN (38%) after day 9	Sig. improved survival and 3-month HRQoL with improved nutrition delivery
Secondary outcome	Sig. higher infectious complications, duration of MV, and hospital LOS for early PN	Full feeding group more likely to be discharged home than rehabilitation unit (*p *<0.04)	No change in HRQoL at 12 months	No difference in LOS or duration of MV	Sig. shorter duration of MV	Longer duration of MV and ICU LOS, and higher infection rate for goal calorie study group	No sig. difference in the ICU LOS, hospital LOS, or mortality	Sig. improvement in HRQoL in MICU patients at 3 and 6 months with improved nutrition delivery
					Improved HRQoL for early PN group			
					No change in infection in PN vs. EN			
Protein delivery	0.8 g/kg/day in all patients	0.8 g/kg/day in both groups (including full feed group)	0.6-0.8 g/kg/day in both groups (including full feed group)	0.6 g/kg/day in all patients	1.1-1.2 g/kg/day in early PN group	>1.0 g/kg/day in supplemental PN group	1.0-1.1 g/kg/day in supplemental PN group	Observational trial
								Protein intake not able to be quantified

All times for length of stays and mechanical ventilation presented in days

*Sig*. significant, *HRQoL *health-related quality of life, *LOS *length of stay, *MV *mechanical ventilation, *PN *parenteral nutrition; *RCT *randomized controlled trial

Whereas the trials not reaching a protein delivery of > 1.0 g/kg/d consistently show no benefit of additional nutrition support versus trophic or permissive underfeeding in the ICU. As protein is a fundamental building block of lean body mass, it will be vital to include protein delivery as a measure in nutrition intervention studies evaluating QOL.

A great challenge to delivering adequate protein is the insufficient protein content of most commercial EN feeds. As a result, survey data have shown that ICU caregivers worldwide deliver on average 0.6 g/kg/day protein, not just for the first few days of ICU stay but for the first 12 days or longer [34]. As a result, meeting protein goals via EN alone has not been successful. Given this, PN should be considered sooner in higher risk ICU patients to assist in meeting calorie and protein needs. Three recent large trials of both supplemental and full total parenteral nutrition (TPN) support versus EN in the ICU setting have shown that TPN use in the ICU is no longer associated with increased infection risk [19-21].

In summary, the risk of trophic or permissive feeding in the first week of the ICU stay should not be considered safe or indicated in higher risk ICU patients, because it has been associated with increased mortality and impairment of long-term QOL. The greater concern is that currently we are unable to predict who will prove to be the "high risk" patients--those who ultimately require prolonged mechanical ventilation and become the "long stayers". Thus, any wide recommendation for trophic or permissive underfeeding during the first week of ICU stay may lead to harm in long-staying ICU patients, who will only reveal themselves when it is too late to make-up the calorie and protein debt they have acquired during their first ICU week [30]. In addition, trials examining the role of independent parenteral administration of amino acids/protein have recently been completed or are underway to help address whether isolated protein delivery can improve outcome, particularly when combined with exercise.

## Is optimal nutrition delivery enough to optimize post-ICU quality of life?

**Figure 4:**
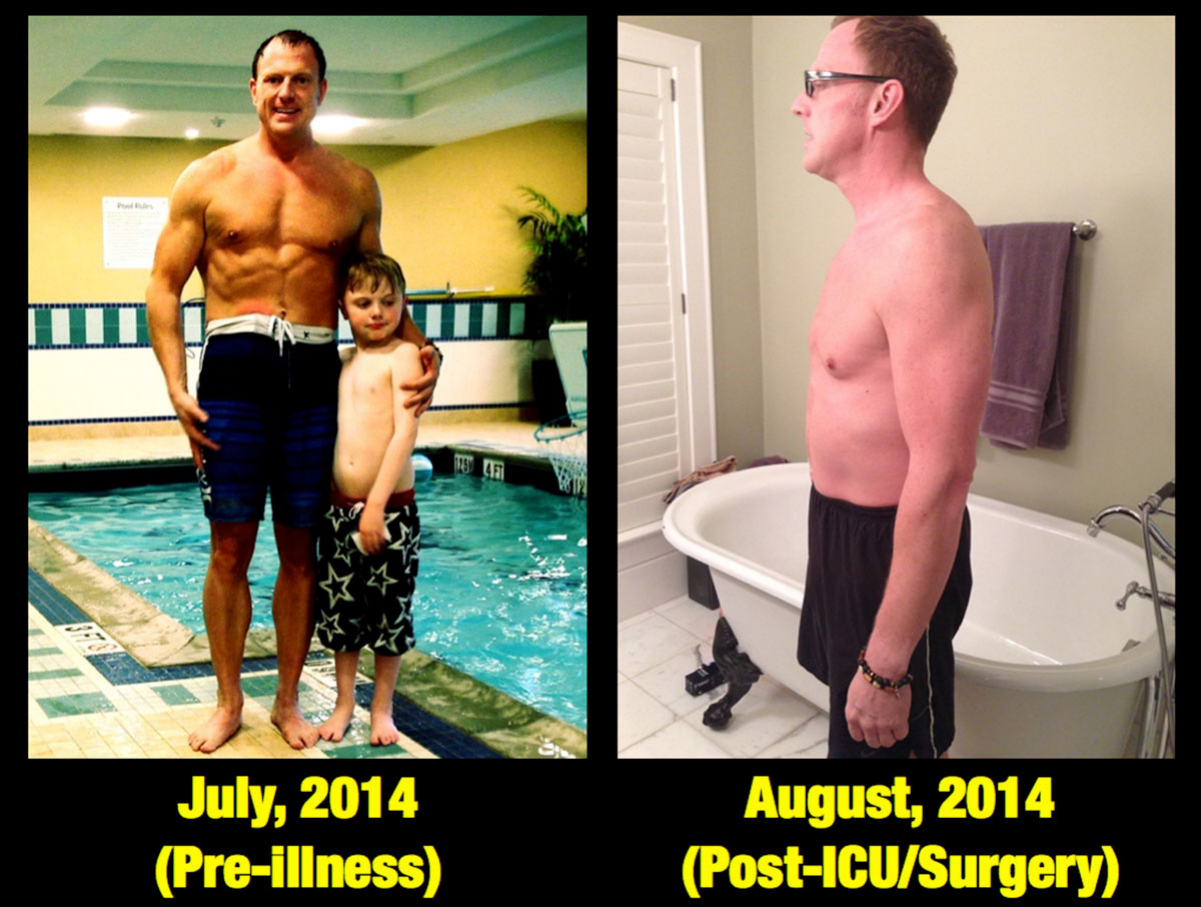
**Lean Body Mass Loss Over 20 days following surgery and critical illness (20 kg over 20 days = 1 kg lean body mass lost/day)**.

The primary author of this article (PEW) has long wondered whether nutritional delivery alone during acute illness was sufficient to optimize preservation of lean body mass and recover QOL and physical function--and recently experienced first hand that the answer is no. This is based on personal experience with nutrition, which began at age 15 when I was diagnosed with ulcerative colitis and lost 30 kg of body weight over 8 weeks on TPN. Although undoubtedly TPN saved my life, as it has saved many lives over the last 40 years, it alone was not able to prevent a major loss of lean body mass and strength in the face of a severe inflammatory injury and multiple operations. I experienced this once again in summer 2014, when I was in perhaps the best physical condition of my life (Figure 4). In August 2014 I suffered a complicated bowel obstruction leading to massive bowel edema and an emergent operation that led to a brief ICU stay and prolonged hospital stay. During this 23-day postoperative course I lost 20 kg body weight despite being on PN or oral feeding supplements for the entire period. At discharge, I had lost significant lean body mass and was not able to walk down the hospital hallway without becoming short of breath. It took 8 months to recover this weight, and for the first 3 months I was not even strong enough to pick up my 5-year-old son.

Without doubt, calorie and protein delivery is mandatory to allow for recovery, both clinically and functionally, but it alone will not optimize our patients' chances to hold their children again. As I have stated in past discussions [35], we as humans are not evolved to survive major critical or surgical illness. Mother Nature never intended us to survive major trauma from the saber-tooth tiger attack on the caveman, and similarly we are not evolved to survive major trauma, sepsis, or surgical interventions. Although we may save many patients using modern technology and get them out of the ICU, our lean body mass reserve and overall metabolic reserve are not sufficient in many cases to allow for a meaningful QOL again. As previously discussed, hypermetabolism and catabolism can persist for months to years after illness/injury [13,14] and this will require not only optimal nutrition, but perhaps pharmacologic intervention to overcome.

## Lessons from elite athletes: role of anabolic and anticatabolic agents

To optimize the G in Figure 1, Gaining function and Growing muscle, we can again look for guidance to basic physiology and to our elite athletes who are masters at gaining and retaining lean body mass. Elite athletes in many sports have proven again and again the benefits of anabolic and anticatabolic agents that are now beginning to be employed successfully in acute illness.

A growing body of literature is beginning to show the benefits of anticatabolic agents such as beta-blockers in burn injury and sepsis [36,37]. Dr Herndon was the first to demonstrate that even a severely burned child could be made anabolic in the face of a major catabolic stress by the administration of propranolol [38]. This intervention also reduced hypermetabolism in this otherwise intractable catabolic injury. Further, septic shock patients who remain tachycardic following resuscitation (perhaps a genetic or evolutionary hypermetabolic tendency in some patients?) can have their mortality reduced 40% by esmolol administration [37]. This is an intervention that has now been shown to be safe in a number of ICU patient populations that may finally be able to reverse the persistent hypermetabolism we have "mis-evolved" in modern ICU care. Future studies are desperately needed focusing on QOL life following beta-blocker administration.

**Figure 5:**
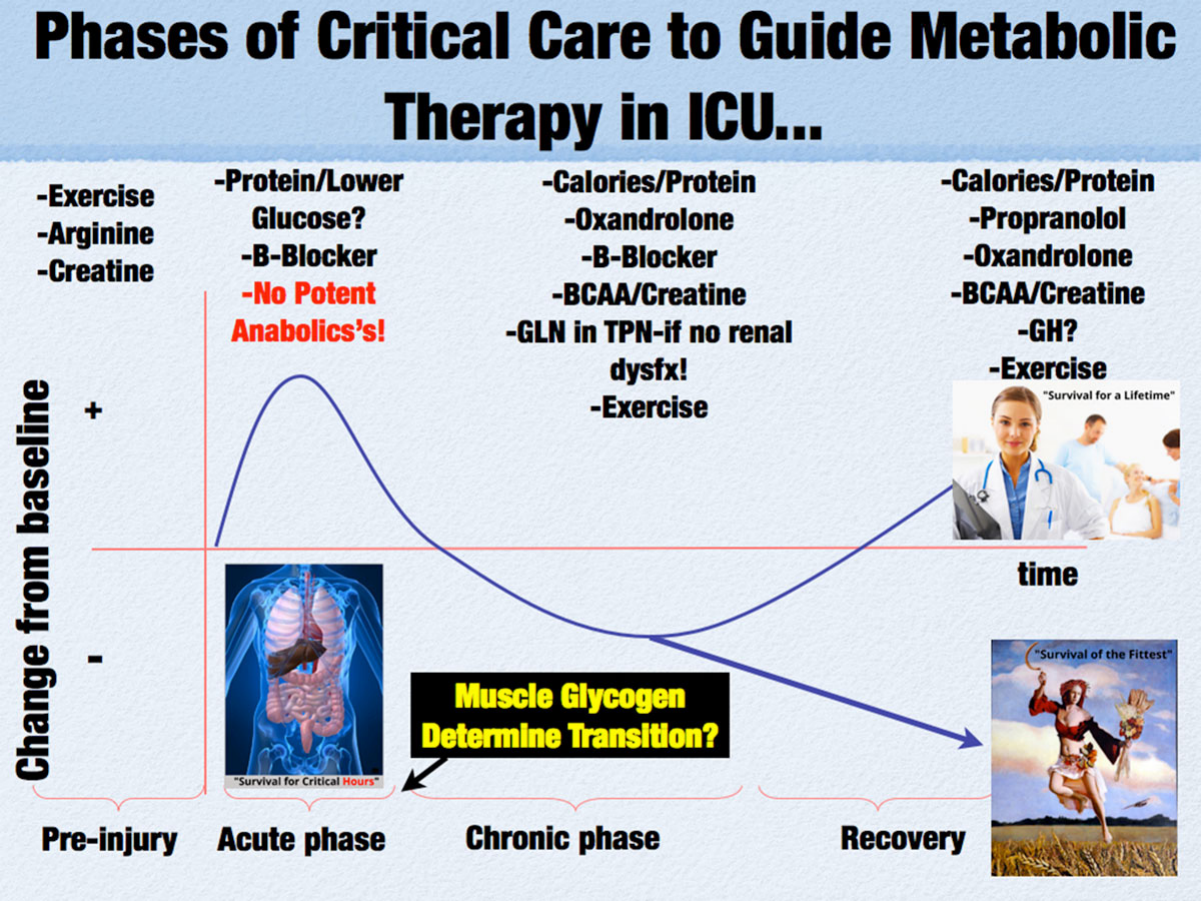
**Phases of Critical Care and Metabolic Therapy in ICU**. BCAA-Branch Chain Amino Acids. Dysfx-Dysfunction. GH-Growth Hormone. GLN-Glutamine. TPN-Total Parenteral Nutrition

Further, anabolic agents such as oxandrolone have shown to be efficacious in reducing the length of stay, shortening time to wound healing, and improving survival in major burns [36]. These agents unquestionably improve lean body mass and function in both patients and athletes. However, the question remaining unanswered is when to initiate them? Ideally, these agents (oxandrolone, Growth Hormone, etc.) would be initiated following the transition from the "acute phase" to the recovery phase [35] (see Figure 5). An objective measurement predicting this transition has yet to be described; we would like to hypothesize that a measure of muscle health, like the muscle glycogen test described previously, could one day be a measure to signal this transition. We noted that a number of patients showed recovery of their muscle glycogen (with scores increasing from 0 to >15) over the first week of ICU stay. When muscle glycogen scores begin to increase, we hypothesize patients may be able to sustain anabolism and be responsive to an anabolic agent like oxandrolone (Figure 5).

## The role of exercise: can we learn from our elite athletes and teach our patients to exercise "smarter"?

**Figure 6:**
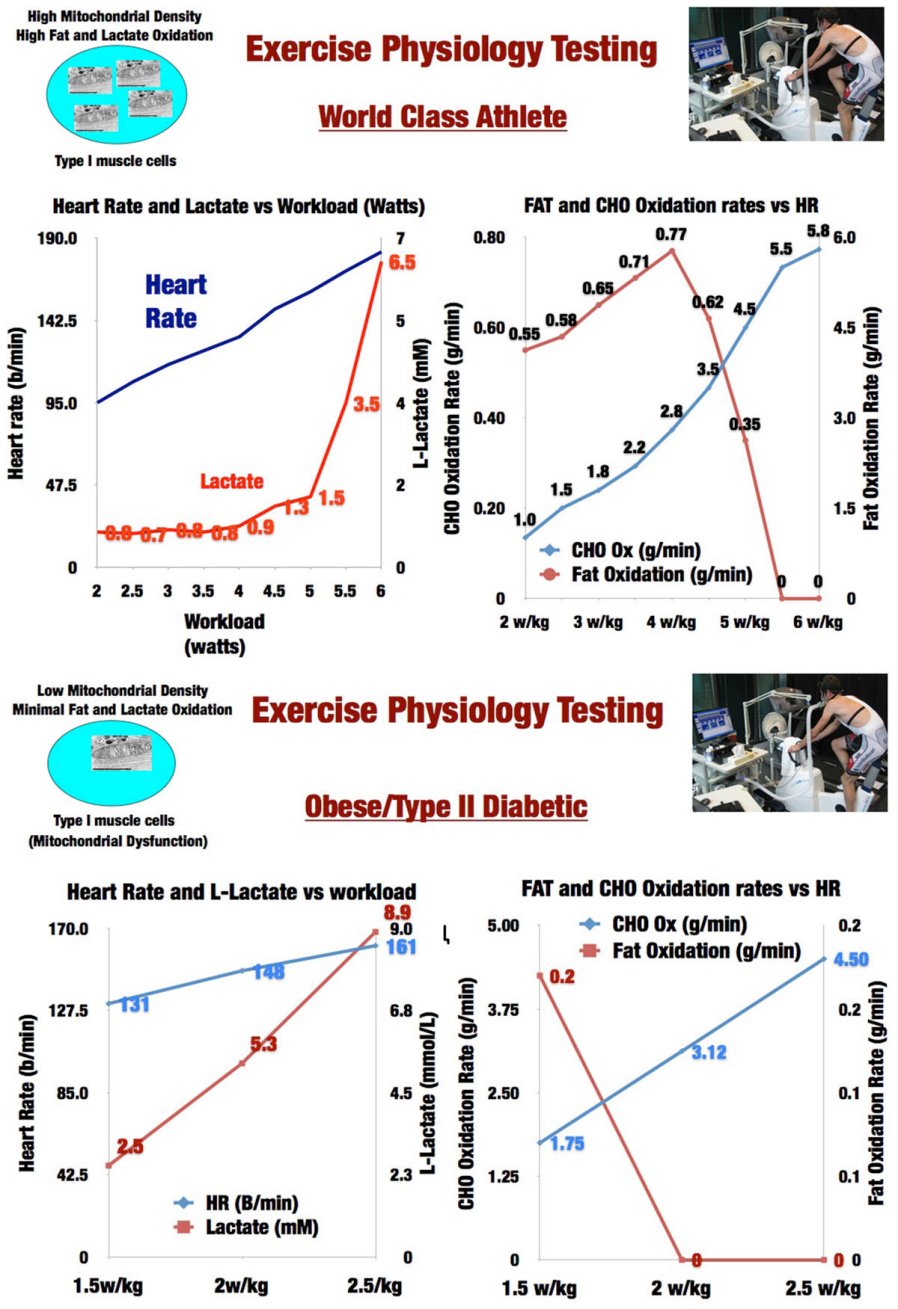
**a: Exercise Physiology Testing in World Class Athlete**. b: Exercise Physiology Testing in Obesity/Type 2 Diabetes. CHO-Carbohydrate. HR-Heart Rate.

Optimal nutrition and anticatabolic/anabolic agents will only be fully successful in improving post-ICU QOL if we learn to maximize the use and benefit of exercise in our ICU survivors. Without question, promoting early mobility improves post-ICU QOL [39]. But can we learn from our most elite athletes how to diagnose ICU-induced exercise and muscle mitochondrial dysfunction and teach our patients how to objectively maximize the benefits of their exercise efforts? Mitochondrial dysfunction has been observed in septic patients and is associated with severity and outcome in septic shock and in the pathogenesis of multiorgan dysfunction syndrome [40,41]. Moreover, it is well established that mitochondrial dysfunction is at the center of the pathogenesis of insulin resistance (IR) and type 2 diabetes mellitus (T2DM) [42,43]. This condition causes important metabolic dysregulation and metabolic inflexibility due to the inability to oxidize fat and CHO properly [44-46], as we have observed in our exercise physiology laboratory (Figure 6b). On the opposite end of the metabolic spectrum, elite endurance athletes possess the most developed mitochondria of any humans, which is key for athletic performance (Figure 6a) [47,48]. Elite athletes in the Tour De France and other extreme athletic endeavors have utilized advanced metabolic and physiological testing and monitoring (such VO_2 _max, (**maximum **volume of oxygen that a human can use. It is measured in millilitres per kilogramme of body weight per minute (ml/kg/min).)

lactate metabolism, and threshold, as well as substrate-use optimization testing) to target and individualize the ideal exercise intensities and workload levels they should be training at to optimize performance. Why should ICU patients not have access to the same methodologies?

Modern exercise physiology testing and monitoring as optimized and routinely performed by expert exercise physiologists, such as the second author of this article (IS-M), allows for the accurate determination of mitochondrial function and substrate utilization by muscle at increasing workloads (see Figure 6). This allows for an ideal and individualized exercise intensity (i.e., heart rate) and workload to be targeted to increase mitochondrial function and performance efficiency in the forms of increased lactate clearance capacity and capacity to oxidize fat in the muscle (Figure 6a), which is key for performance. This is the same kind of endurance and function that will allow our ICU survivors to walk up the stairs or walk down the street again. Muscle depends on both glucose and fat to function. Humans have a quite limited glycogen reserve (400-600 g) and must then rely on fat metabolism for any prolonged exercise or activity. For fat to be used efficiently by the muscle, mitochondrial metabolism must be optimized because fat can only be oxidized to synthesize ATP in the mitochondria via the Krebs cycle (Tricarboxylic Acid Cycle (TCA)) and electron transport chain. Likewise, under resting conditions, most glucose is oxidized in the mitochondria via pyruvate oxidation in the TCA. When excessive glycolytic flux occurs during the stress response (exercise/critical illness) pyruvate cannot be fully oxidized to acetyl-CoA for ATP synthesis in the mitochondria. Pyruvate dehydrogenase (PDH) saturates and drives the reduction of pyruvate to lactate increasing blood lactate levels. During high-intensity exercise, glycolysis is very elevated due to necessity to synthesize ATP in a fast manner as is elicited by the critical illness stress response. Therefore, increased lactate production and hyperlactatetemia as observed in critical illness are the norm in many forms of athletic competition. The important difference with ICU patients is that: well-trained athletes have an excellent lactate clearance capacity [48,49] due to great mitochondrial capacity, allowing them to continue performing high-intensity work; and exercise activity ceases at some point when competition finishes, restoring cellular metabolism back to basal levels. However, none of this happens in the ICU because the "race" for survival is 24/7. This is further complicated by mitochondrial dysfunction, which results in decreased lactate clearance capacity as has been described and correlated with ICU survival [50].

**Figure 7:**
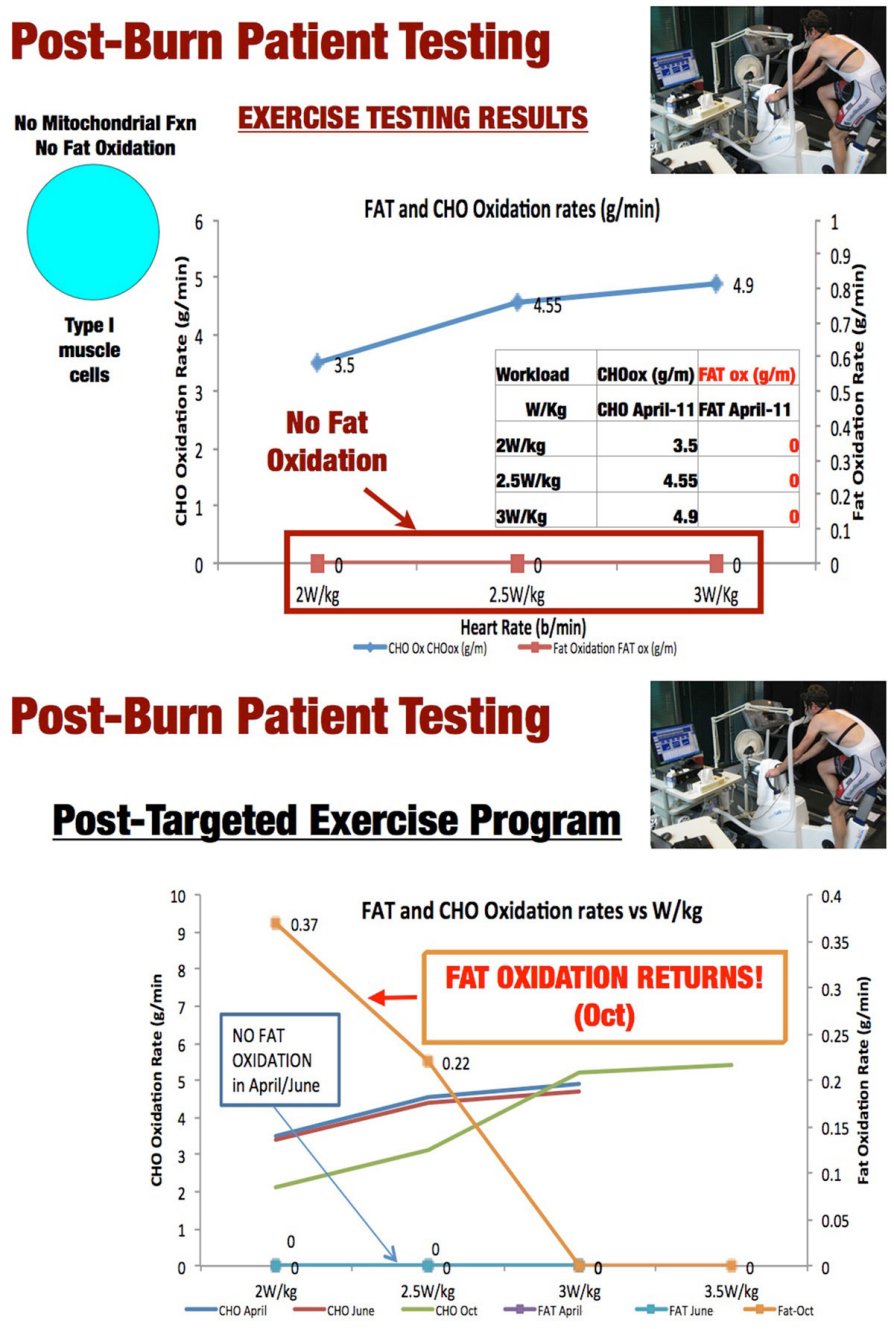
**A - Exercise Physiology Testing in Burn Patient Prior to Metabolic and Exercise Therapy**. B - Exercise Physiology Testing in Burn Patient Following Metabolic and Exercise Therapy. CHO-Carbohydrate. Ox-Oxidation.

It is likely that mitochondrial dysfunction continues for a prolonged period of time post ICU, contributing to increased morbidity and decreased lifespan. We have recently demonstrated this in a patient who presented to our exercise physiology clinic 6 months following discharge from our burn unit (key identifying information changed to protect patient identity) [51]. This patient was a 49-year-old male who presented with a 35% Total Body Surface Area (TBSA) burn injury. He was in our burn ICU for 24 days and made a full and uncomplicated recovery. He had been an active individual prior to his injury who enjoyed biking and running. Three months after hospital discharge he attempted to return to biking and found that he could not even cycle for 5 minutes without becoming quite exhausted, forcing him to get off his bike and rest for a prolonged period of time before he was able to bike again, and then only for a few minutes maximum. He attempted to improve his endurance for a number of months with no success at which he point he presented to Dr Inigo San-Millan and our Exercise Physiology Clinic at the University of Colorado. Exercise physiology testing was performed that showed something quite remarkable, and disturbing. This young man, 6 months out from his moderate to severe burn injury, had absolutely no capacity to utilize fat for energy in his muscle (Figure 7a). This severe deficit had never been observed in our clinic during testing of many thousands of subjects. The physiologic testing was repeated a month later and the same results were obtained. This finding was found to be consistent with "exercise metabolic dysfunction" or muscle mitochondrial dysfunction. Therefore, we believe that in the same manner as elite endurance athletes improve their mitochondrial function through specific and individualized training based on individualized heart rate zones, power zones, or speed paces, targeted individualized exercise prescription/training through heart rate monitoring could lead to optimization of physical therapy efforts as part of a "metabolic rehabilitation" for patients during and after an ICU stay.

We have discovered that our methodology and technology can not only target training, but also diagnose post-illness muscle dysfunction that can lead to serious impairment of QOL recovery. We applied our methodology with the patient described previously who was placed on an individualized training plan for 4 months with specific training zones in the same manner as we apply to elite athletes. On retesting 4 months later his ability to utilize fat as a substrate in his muscle had returned and his heart rate training target adjusted upward (Figure 7b). Within 1 year following initiation of training, the patient was fully recovered and competing in >100 km bike races.

The vital question this raises is how many of our post-ICU "victims" suffer from this sort of exercise metabolic dysfunction and mitochondrial dysfunction? It also leads to a hypothesis that without this targeted performance testing (available to anyone at many health clubs and university athletic departments), would any of these patients (including the burn patient described here) ever recover? Perhaps this is one explanation for the data of Herridge et al. [8] showing that the physical QOL impairments observed following an ICU stay often do not improve even after 5 years or more following hospital discharge. This testing can be initiated to guide physical therapy and exercise while a patient is still ventilated (perhaps using in-bed ergometers) and continued throughout the recovery period to give our ICU patients the best chance to be "survivors" instead of "victims".

## What can we do to start winning war and create survivors and not victims after ICU?

In summary, the often "Pyrrhic Victories" of the past must not be accepted in the future of ICU care. Getting patients out of the ICU is not enough! We can no longer take comfort in the improved ICU survival of our patients when a rapidly growing number will leave to a nursing home or rehabilitation center where many will meet their end, never able to walk or hold their grandchildren again. If we cannot learn to address this post-ICU QOL loss or post-ICU syndrome, one might ask why we practice intensive care at all? We must learn to ask our patients what are their goals/reasons for enduring ICU care (perhaps if only to take an evening stroll with their wife or husband again) and make meeting these goals by optimizing post-ICU QOL a priority from the moment they are admitted.

We are learning that this may be as easy as A ... B ... C ... D ... E ... F ... G ... (Figure 1). But we must pay attention to these key care pathways from the day of admission until after discharge. When considering F and G, nutrition and protein delivery are fundamental to optimal post-ICU QOL of life. But, to optimize recovery of QOL, targeted evaluation of the nutritional state (via the NUTRIC score), lean body mass, and muscle health (via ultrasound and other modalities) will also be required. Further, interventions used by athletes worldwide, such as anabolic/anticatabolic therapy and exercise physiology testing, must be studied further and implementation projects performed. Again, why should only our "healthiest" patients benefit and have access to our most advanced recovery technologies, when our ICU patients need and are likely to benefit more from these innovations than any other "patient"? In closing, if we are to start creating survivors and not victims following an ICU stay, we must go to any length to liberate them from Mother Nature's desire to prevent their recovery and utilize all existing innovations to help them win the war!

## Abbreviations

ARDS, Acute respiratory distress syndrome; CI, Confidence interval; CID, Clinical important difference; CT, Computed tomography; EN, Enteral nutrition; HR, Hazard ratio; ICU-AW, ICU-acquired weakness; IR, Insulin resistance; MICU, Medical ICU; MRI, Magnetic resonance imaging; PDH, Pyruvate dehydrogenase; PN, Parenteral nutrition; QOL, Quality of life; SF-36, Short Form-36; T2DM, Type 2 diabetes mellitus; TPN, Total parenteral nutrition.

## Competing interests

IS-M is co-founder of MuscleSound. PEW receives research grants from National Institutes for Health (this work supported by National Heart Lung and Blood Institute (NHLBI) R34 HL109369), Canadian Institutes for Health Research, and American Burn Association; receives research grants from and is a speaker and consultant for Fresenius Inc., Abbott Inc., and Nestle Inc.; is a speaker for Baxter Inc.; is a consultant for Lyric Pharmaceuticals, Theravance Inc., and Covidian Inc.; receives research grants from GSK; and is a speaker for Nutricia Inc.

## Supplementary Material

Additional file 1 video #1 used with permission of Wes Ely and http://www.icudelerium.org. Click here for file

Additional file 2 video #2 used with permission of Wes Ely and http://www.icudelerium.org. Click here for file
